# Prioritizing genes for follow-up from genome wide association studies using information on gene expression in tissues relevant for type 2 diabetes mellitus

**DOI:** 10.1186/1755-8794-2-72

**Published:** 2009-12-31

**Authors:** Hemang Parikh, Valeriya Lyssenko, Leif C Groop

**Affiliations:** 1 Department of Clinical Sciences, Diabetes and Endocrinology, Lund University, University Hospital Malmö, Malmö, Sweden; 2 Laboratory of Translational Genomics, Division of Cancer Epidemiology and Genetics, National Cancer Institute, National Institutes of Health, Bethesda, Maryland, USA; 3 Department of Medicine, Helsinki University, Helsinki, Finland

## Abstract

**Background:**

Genome-wide association studies (GWAS) have emerged as a powerful approach for identifying susceptibility loci associated with polygenetic diseases such as type 2 diabetes mellitus (T2DM). However, it is still a daunting task to prioritize single nucleotide polymorphisms (SNPs) from GWAS for further replication in different population. Several recent studies have shown that genetic variation often affects gene-expression at proximal (*cis*) as well as distal (*trans*) genomic locations by different mechanisms such as altering rate of transcription or splicing or transcript stability.

**Methods:**

To prioritize SNPs from GWAS, we combined results from two GWAS related to T2DM, the Diabetes Genetics Initiative (DGI) and the Wellcome Trust Case Control Consortium (WTCCC), with genome-wide expression data from pancreas, adipose tissue, liver and skeletal muscle of individuals with or without T2DM or animal models thereof to identify T2DM susceptibility loci.

**Results:**

We identified 1,170 SNPs associated with T2DM with *P *< 0.05 in both GWAS and 243 genes that were located in the vicinity of these SNPs. Out of these 243 genes, we identified 115 differentially expressed in publicly available gene expression profiling data. Notably five of them, *IGF2BP2*, *KCNJ11*, *NOTCH2*, *TCF7L2 *and *TSPAN8*, have subsequently been shown to be associated with T2DM in different populations. To provide further validation of our approach, we reversed the approach and started with 26 known SNPs associated with T2DM and related traits. We could show that 12 (57%) (*HHEX*, *HNF1B*, *IGF2BP2*, *IRS1*, *KCNJ11*, *KCNQ1*, *NOTCH2*, *PPARG*, *TCF7L2*, *THADA*, *TSPAN8 *and *WFS1*) out of 21 genes located in vicinity of these SNPs were showing aberrant expression in T2DM from the gene expression profiling studies.

**Conclusions:**

Utilizing of gene expression profiling data from different tissues of individuals with or without T2DM or animal models thereof is a powerful tool for prioritizing SNPs from WGAS for further replication studies.

## Background

Genome-wide association study (GWAS) offers unbiased ways to examine association of more than a million single nucleotide polymorphisms (SNPs) with disease [1]. Several GWAS have indentified novel genomic regions influencing risk for type 2 diabetes mellitus (T2DM) [2-6]. However, the challenge remains to prioritize SNPs from GWAS for further replication [1]; even if one would try to replicate only the top 1% with strongest genetic evidence 5,000-10,000 SNPs need to be genotyped in replication studies. Most available methods for prioritizing genes for follow-up from GWAS are based upon bibliometric analyses such as Gene Relationships Among Implicated Loci (GRAIL) [7]. In addition, bioinformatic tool such as TEAM (a tool for the integration of expression and linkage and association maps) is designed to integrate linkage, association and expression data, together with functional annotations however currently it is not implemented to handle GWAS data [8].

Genetic variation often influences gene expression by different mechanisms such as altering rate of transcription or splicing or transcript stability [9]. In this study we first have used published gene expression profiling data in key tissues (*i.e*. pancreas, adipose tissue, liver and skeletal muscle) from humans and animal models of T2DM to identify genes in GWAS which would deserve follow-up in replication studies. Secondly to provide further validation of our approach, we reversed the approach and tested 21 genes (*ADAMTS9*, *CDKAL1*, *CDKN2B*, *FTO*, *GCK*, *GCKR*, *HHEX*, *HNF1B*, *IGF2BP2*, *IRS1*, *JAZF1*, *KCNJ11*, *KCNQ1*, *MTNR1B*, *NOTCH2*, *PPARG*, *SLC30A8*, *TCF7L2*, *THADA*, *TSPAN8 *and *WFS1*) located in vicinity of 26 known SNPs associated with T2DM and related traits [2,5,6,10-13] for their expression in the same data sets.

## Methods

Results from two separate GWAS are reported here.

### Diabetes Genetics Initiative (DGI)

The DGI included 1,464 T2DM patients and 1,467 normoglycemic controls from Sweden and Finland [2]. These samples were genotyped on the Affymetrix GeneChip^® ^Human Mapping 500K Array Set which contains ~500,000 SNPs for interrogation. 386,731 autosomal SNPs were included for further analyses which have genotype call frequency > 0.95, the Hardy-Weinberg equilibrium (HWE) *P *> 10^-6 ^in controls and a minor allele frequency (MAF) > 0.01 in both populations.

### Wellcome Trust Case Control Consortium (WTCCC)

The WTCCC included 1,924 T2DM patients and 2,938 population controls from UK (6). These samples were also genotyped on the Affymetrix GeneChip^® ^Human Mapping 500K Array Set which contains ~500,000 SNPs for interrogation. 393,453 autosomal SNPs were included for further analyses which have genotype call frequency > 0.95, the HWE *P *> 10^-4 ^in the total sample and a MAF > 0.01.

### Meta-analysis of T2DM and related traits GWAS

Recently large scale meta-analysis of T2DM and related traits GWAS comprised tens of thousands of individuals and investigated ~2.2 million SNPs (directly genotyped and imputed), followed by well-powered replication studies have robustly identified a total of 26 SNPs (rs4607103, rs12779790, rs7754840, rs10811661, rs2191349, rs9939609, rs560887, rs4607517, rs780094, rs1111875, rs4430796, rs757210, rs1470579, rs2943641, rs7578326, rs864745, rs5219, rs2237892, rs10830963, rs10923931, rs1801282, rs13266634, rs7903146, rs7578597, rs7961581 and rs10010131) located in vicinity of 21 genes (*ADAMTS9*, *CDKAL1*, *CDKN2B*, *FTO*, *GCK*, *GCKR*, *HHEX*, *HNF1B*, *IGF2BP2*, *IRS1*, *JAZF1*, *KCNJ11*, *KCNQ1*, *MTNR1B*, *NOTCH2*, *PPARG*, *SLC30A8*, *TCF7L2*, *THADA*, *TSPAN8 *and *WFS1*) been associated with T2DM and related traits in several population [2,5,6,10-13].

### Gene expression profiling data

Results from five gene expression profiling studies from four different tissues (*i.e*., pancreas, adipose tissue, liver and skeletal muscle) in humans and animal models have been used for the analysis of the putative affect of a SNP on gene expression.

#### (a) From pancreas

##### (1) Pancreatic islets from healthy controls and T2DM patients

We included gene expression profiling data of human islets from cadaver islet donors with and without T2DM [14]. We downloaded the raw gene expression data from the Diabetes Genome Anatomy Project (DGAP) database, accession number 103 http://www.diabetesgenome.org/chipperdb/expt.cgi?id=103.

##### (2) Pancreas from healthy and diabetic rats

We also evaluated the effect of streptozotocin (STZ) induced diabetes on expression of genes of interest in pancreas using previously published microarray data [15]. We downloaded the raw gene expression data from diabetic and healthy untreated rats from the National Center for Biotechnology Information's (NCBI) Gene Expression Omnibus (GEO) database, accession number GSE2470 http://www.ncbi.nlm.nih.gov/geo/query/acc.cgi?acc=GSE2470.

#### (b) From skeletal muscle

##### (3) Skeletal muscle from healthy controls and T2DM patients

We analyzed the expression of individual genes using our previously published microarray data from human skeletal muscle of healthy controls and T2DM patients [16]. We downloaded the raw gene expression data from the DGAP database, accession number 54 http://www.diabetesgenome.org/chipperdb/expt.cgi?id=54

##### (4) Skeletal muscle from healthy and diabetic mice

We evaluated the effect of STZ- induced diabetes on expression of genes of interest in skeletal muscle from previously published microarray data [17]. We downloaded the raw gene expression data from diabetic and healthy mice from the DGAP database, accession number 104 http://www.diabetesgenome.org/chipperdb/expt.cgi?id=104

#### (c) From adipose tissue, liver and skeletal muscle

##### (5) From Zucker diabetic fatty (ZDF) and Zucker lean control (ZLC) rats

Finally, we also examined the expression of genes of interest in insulin-sensitive tissues like adipose tissue, liver and skeletal muscle of ZDF rats at age of 6 weeks (pre-diabetic) and 12 weeks (diabetic) and compared with age- and sex-matched ZLC rats [18]. We downloaded the normalized gene expression data from the NCBI's GEO database, accession number GSE1080 http://www.ncbi.nlm.nih.gov/geo/query/acc.cgi?acc=GSE1080

All studies were conducted according to the principles of the Helsinki Declaration and approved by respective local ethics committees [2,5,6,10-18].

### Statistical Analysis

#### SNPs and gene selection

We used the NCBI's single nucleotide polymorphism database (dbSNP) build 130 to identify genes located in the vicinity of selected SNPs. Homologues of the genes for mouse and rat were identified using the NCBI's HomoloGene release 64. We included only those genes that were evolutionarily conserved in three different species namely human, mouse and rat.

#### Analysis of microarray data

We used ENTREZ custom chip definition files to regroup the individual probes into consistent probesets and remap them to the correct sets of genes for different Affymetrix arrays [19-21]. The method used for calculating gene expression from Affymetrix array data can have a major impact on the results [22-24]. Hence, we used four different procedures for normalization and summarization which combines the multiple probe intensities for each gene to produce an expression value: (1) by MAS5.0 algorithms to adjust for background noise level using estimates of the distribution of probe intensities, scaling based normalization and summarization based on a Tukey's biweight [25], (2) by the GC-content Robust Multi-array Average (GC-RMA) method [26] with additional background adjustment using sequence information to estimate probe affinity for nonspecific binding, quantile based normalization and summarization based on a multi-array model fit using median polish algorithm, (3) by Probe Logarithmic Intensity Error (PLIER) method (Affymetrix) utilizing both perfect match and mismatch signaling with quantile based normalization and (4) by Robust Multi-array Average (RMA) method [27,28] which implements model-based background adjustment, quantile based normalization and summarization based on a multi-array model fit using median polish algorithm.

For interclass, unpaired comparisons, we used a two-tailed Student's *t*-test with equal variance to identify differences in expression (log (base 2) transformed) of individual gene from different gene expression profiling studies (for each of four normalization methods namely MAS5.0, GC-RMA, PLIER and RMA discretely). Due to hypothesis generating nature of this study, we considered only those genes that were significantly altered in diabetes or associated traits with a *P *< 0.05 in at least one normalization method. For the ZDF and ZLC rat studies, we used fold change values which were calculated by dividing the median of normalized signal channel intensity (Cy5) by the median of normalized control channel intensity (Cy3) to assess the difference between ZDF vs. ZLC. The genes with expression ratios (*i.e*., ratio of normalized mean signal intensities of ZDF rat gene to that of ZLC) lesser than 0.67 or greater than 1.50 were considered differentially expressed.

## Results

**Figure 1:**
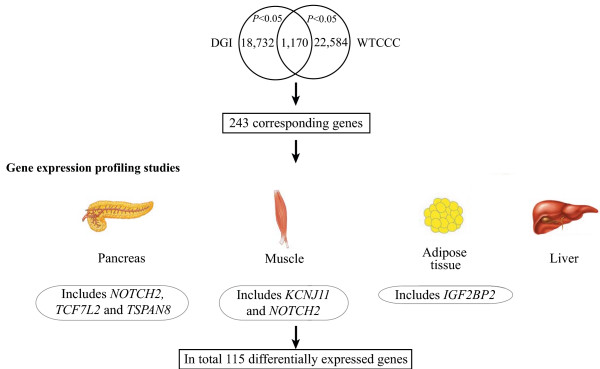
**Schematic diagram of prioritizing SNPs from GWAS**. 1,170 SNPs were associated with T2DM with *P *< 0.05 in both GWAS and 243 genes were located in vicinity of these SNPs. Out of these 243 genes, 115 were differentially expressed between diabetic and healthy tissues in different gene expression profiling studies.

To identify T2DM susceptibility loci, we compiled results from two previously reported GWAS which used the Affymetrix GeneChip^® ^Human Mapping 500K Array Set containing ~500,000 SNPs for interrogation [2,6]. To provide a framework for the analytical approach, we have used different cut-off of *P*-values from both GWAS (Table 1). For instance, we selected 1,170 directly genotyped SNPs associated with T2DM with *P *< 0.05 in both GWAS (Table 1) and 243 genes were located in the vicinity of these SNPs (Table 2). To determine whether any of these genes exhibit altered expression in diabetes or associated traits in humans or rodent organisms, we utilized published gene expression profiling data from pancreas, adipose tissue, liver and skeletal muscle (Methods). Out of 243 genes, we identified 115 genes differentially expressed between diabetic and healthy tissues in these studies (Additional file 1: Supplemental Table S1, Tables 3 and 4 and Figure 1). Out of 115 genes, we identified five genes (namely *IGF2BP2*, *KCNJ11*, *NOTCH2*, *TCF7L2 *and *TSPAN8*) for which SNPs located in their vicinity have shown association with T2DM in different populations [2,5,6]. Moreover, the results for SNPs selected based on different GWAS *P*-values thresholds are shown in Table 1 to Table 4.

**Table 1:** Numbers of SNPs associated with T2DM with different cut-off of *P*-values in DGI and WTCCC GWAS

	*P *< 0.01 (DGI)	*P *< 0.05 (DGI)
***P *< 0.01 (WTCCC)**	61	299

***P *< 0.05 (WTCCC)**	215	1,170

***P *< 0.01 in DGI and *P *< 0.05 in WTCCC or** ***P *< 0.01 in WTCCC and *P *< 0.05 in DGI**	453	

**Table 2:** Numbers of genes located in the vicinity of the SNPs (from Table 1) which are associated with T2DM with different cut-off of *P*-values in DGI and WTCCC GWAS

	*P *< 0.01 (DGI)	*P *< 0.05 (DGI)
***P *< 0.01 (WTCCC)**	18	86

***P *< 0.05 (WTCCC)**	52	243

***P *< 0.01 in DGI and *P *< 0.05 in WTCCC or** ***P *< 0.01 in WTCCC and *P *< 0.05 in DGI**	118	

**Table 3:** Numbers of genes for which expression levels in pancreas, skeletal muscle, adipose tissue or liver were altered in diabetes as compared to controls

	*P *< 0.01 (DGI)	*P *< 0.05 (DGI)
***P *< 0.01 (WTCCC)**	11	42

***P *< 0.05 (WTCCC)**	30	115

***P *< 0.01 in DGI and *P *< 0.05 in WTCCC or ** ***P *< 0.01 in WTCCC and *P *< 0.05 in DGI**	60	

**Table 4:** Numbers of genes (out of the genes from Table 3) for which SNPs located in their vicinity have shown association with T2DM in different populations [2,5,6]

	*P *< 0.01 (DGI)	*P *< 0.05 (DGI)
***P *< 0.01 (WTCCC)**	2 *(OR = 1.31, *P *= 1)	5 *(OR = 2.81, *P *= 0.26)

***P *< 0.05 (WTCCC)**	2 *(OR = 1.49, *P *= 1)	5 *(OR = 2.85, *P *= 0.26)

***P *< 0.01 in DGI and *P *< 0.05 in WTCCC or** ***P *< 0.01 in WTCCC and *P *< 0.05 in DGI**	5 *(OR = 2.53, *P *= 0.44)	

*OR = Odds ratio, *P*-values refer to a two-tailed Fisher's Exact test.

**Figure 2:**
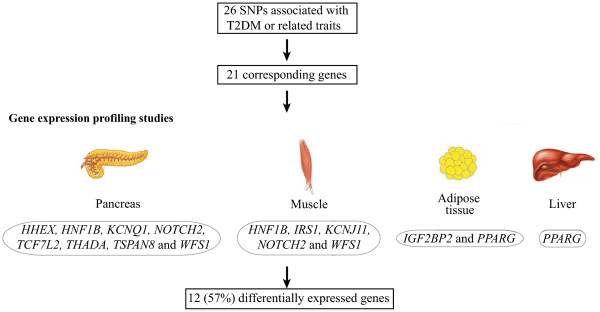
**Identification of differentially expressed genes using gene expression profiling studies from SNPs associated with T2DM and related traits**. 26 SNPs are associated with T2DM and related traits and 21 genes are located in vicinity of these SNPs. Out of these 21 genes, 12 were differentially expressed between diabetic and healthy tissues in different gene expression profiling studies.

While in the first part we used the GWAS studies to confirm the findings from the expression arrays, in the second part we asked the question whether any of the 26 SNPs associated with T2DM or related traits identified through meta-analysis of GWAS would show differences in expression between diabetic and healthy tissues in the same data sets (Methods). Intriguingly, 12 (57%) (*HHEX*, *HNF1B*, *IGF2BP2*, *IRS1*, *KCNJ11*, *KCNQ1*, *NOTCH2*, *PPARG*, *TCF7L2*, *THADA*, *TSPAN8 *and *WFS1*) out of 21 genes located in vicinity of these SNPs were differentially expressed in tissues from T2DM individuals/animal models as compared to healthy tissues (Additional file 1: Supplemental Table S2 and Figure 2). Out of these 12 genes, eight (*HHEX*, *HNF1B*, *KCNQ1*, *NOTCH2*, *TCF7L2*, *THADA*, *TSPAN8 *and *WFS1*) showed differential expression in pancreatic islets, five (*HNF1B*, *IRS1*, *KCNJ11*, *NOTCH2 *and *WFS1*) showed differential expression in skeletal muscle, two (*IGF2BP2 *and *PPARG*) showed differential expression in adipose tissue and only one (*PPARG*) showed differential expression in liver (Additional file 1: Supplemental Table S2). This finding further supports the notion that defects in different tissues contribute to the pathogenesis of T2DM.

## Discussion

The main objective of this study was to test a novel approach for prioritizing of SNPs from GWAS by combining the results from these studies with publicly available genome-wide expression profiling data from key tissues (*i.e*. pancreas, adipose tissue, liver and skeletal muscle) of T2DM. We identified five genes namely *IGF2BP2*, *KCNJ11*, *NOTCH2*, *TCF7L2 *and *TSPAN8 *for which SNPs located in their vicinity have been consistently associated with T2DM in several populations [2,5,6]. More intriguingly, 12 (57%) (*HHEX*, *HNF1B*, *IGF2BP2*, *IRS1*, *KCNJ11*, *KCNQ1*, *NOTCH2*, *PPARG*, *TCF7L2*, *THADA*, *TSPAN8 *and *WFS1*) out of 21 genes located in vicinity of known 26 T2DM or related traits associated SNPs showed different expression in tissues of individuals with or without T2DM or animal models thereof.

Prioritizing SNPs from GWAS for further replication in other populations is a challenging task due to the fact that a large numbers of comparisons are made in parallel. Moreover, effect sizes of individual SNPs are usually very small and would often not reach significance after correction for multiple testing. To address this problem several strategies were applied including meta-analysis of GWAS and bibliometric-bioinformatic approaches like GRAIL [7]. A meta-analysis of GWAS from different populations identified six novel loci associated with T2DM [5]. However, even though top signals are selected based upon the strength of association large scale replication studies are still needed.

GRAIL is a web-tool to examine relationships between genes in different disease associated loci. It is based on finding similarities in the published scientific text among the associated genes. Using GRAIL for the 1,170 SNPs associated with T2DM (Figure 1) we could identify three genes subsequently being associated with T2DM, namely *JAZF1*, *KCNJ11 *and *TCF7L2 *[4]. In comparison, five genes (*IGF2BP2*, *KCNJ11*, *NOTCH2*, *TCF7L2 *and *TSPAN8*) were identified using our current approach. Moreover, our approach can be very useful for designing tissue specific functional studies. Also, a more general approach such as GeneMiner (which is a meta-analysis approach that integrates data of heterogeneous origin *e.g*. DNA microarrays and complementing qualitative data covering several human and mouse tissues related to T2DM) has identified several functional T2DM candidate genes [29]. However, we were able to identify only two (*HNF1B *and *IRS1*) out of the 21 genes which are associated with T2DM and related traits [2,5,6,10-13] using GeneMiner in contrast to 12 (57%) using our approach (Additional file 1: Supplemental Table S2).

Importantly, our findings demonstrate that more than 50% of the genes in which genetic variants have been known to increase risk of T2DM showed altered expression in different tissues. The perturbation was highest, as expected, in pancreatic islets, where eight genes *i.e*. *HHEX*, *HNF1B*, *KCNQ1*, *NOTCH2*, *TCF7L2*, *THADA*, *TSPAN8 *and *WFS1*, showed aberrant expression. All of these genetic loci, apart from the less studied *TSPAN8*, have been implicated in pathways primarily involved in insulin secretion, cell proliferation and regeneration [30]. Of note, genetic variants in the *THADA *and *WFS1 *have recently been shown to impair glucagon-like peptide-1-stimulated insulin secretion [31,32]. Furthermore, many of these loci have also shown effects on insulin sensitivity [33]. In line with this, five genes, *i.e*. *HNF1B*, *IRS1*, *KCNJ11*, *NOTCH2 *and *WFS1*, were also differentially expressed in skeletal muscle. Of all T2DM genes, *IRS1 *seems to have a clear effect on insulin sensitivity; the T2DM-associated allele was associated with decreased IRS1 protein expression as well as reduced phosphatidylinositol-3-kinase-activity and insulin-stimulated glucose uptake in humans [12].

Defects in fat metabolism and excess fat deposition in the abdominal region play an important role in the pathogenesis of T2DM and obesity [34]. Our findings that expression of the *IGFBP2 *and *PPARG*, genes was altered in adipose tissue further supports this notion. *PPARG *is a nuclear receptor that regulates transcription of genes involved in adipogenesis. A common Pro12Ala polymorphism has been associated with decreased transcriptional activity and increased insulin sensitivity and thereby provides protection against T2DM [35]. Additionally, *PPARG *also showed differential expression in the liver, where it regulates a number of genes involved in both glucose and lipid metabolism. These results add further support to a role for variation in these genes in the pathogenesis of T2DM.

There are several issues to consider in the interpretation of the results. In the current study, we have only examined *cis*-regulatory variants and their possible contribution to phenotypic variations leading to T2DM in contrast to *trans*-regulatory variants. Moreover, different SNP alleles in the vicinity of or within a gene may not alter mRNA level or stability and hence further functional studies are needed to evaluate the biological mechanisms associated with variants in model organisms as well as tissue samples. Future studies integrating genome-wide expression profiles from key tissues of T2DM with GWAS mapping are required for identification of SNPs that are associated with variation in gene expression contributing to T2DM [36].

## Conclusions

Taken together these data show the utility of using gene expression profiling studies to prioritize genes from GWAS for further replication efforts.

## Competing interests

The authors declare that they have no competing interests.

## Authors' contributions

HP conceived the project, designed the study, analyzed the microarray data, performed statistical analysis and drafted the manuscript. VL performed statistical analysis and drafted the manuscript. LCG designed the project and supervised all phases of the project including writing of the manuscript.

All authors have read and approved the final manuscript.

## Pre-publication history

The pre-publication history for this paper can be accessed here:

http://www.biomedcentral.com/1755-8794/2/72/prepub

## Supplementary Material

Additional file 1 **Supplemental tables**. Supplemental Table S1: Genes for which expression levels in pancreas, skeletal muscle, adipose tissue or liver were altered in diabetes as compared to controls. The table lists differentially expressed genes between diabetic and healthy tissues in different gene expression profiling studies. Supplemental Table S2: T2DM and related traits associated genes for which expression levels in pancreas, skeletal muscle, adipose tissue or liver were altered in diabetes as compared to controls. The table lists differentially expressed genes between diabetic and healthy tissues in different gene expression profiling studies. Click here for file
