# Supplementary material for: Prioritizing genes for follow-up from genome wide association studies using information on gene expression in tissues relevant for type 2 diabetes mellitus
Source: BMC Med Genomics. 2009 Dec 31;2:72. doi: 10.1186/1755-8794-2-72 (PMC2815699; doi:10.1186/1755-8794-2-72)
Supplement: Additional file 1 — Supplemental tables. Supplemental Table S1: Genes for which expression levels in pancreas, skeletal muscle, adipose tissue or liver were altered in diabetes as compared to controls. The table lists differentially expressed genes between diabetic and healthy tissues in different gene expression profiling studies. Supplemental Table S2: T2DM and related traits associated genes for which expression levels in pancreas, skeletal muscle, adipose tissue or liver were altered in diabetes as compared to controls. The table lists differentially expressed genes between diabetic and healthy tissues in different gene expression profiling studies. [file 1755-8794-2-72-S1.DOCX]

**Supplemental Table S1:** Genes for which expression levels in pancreas, skeletal muscle, adipose tissue or liver were altered in diabetes as compared to controls

1. **NGT vs. T2DM human pancreatic islets**

| **Gene Symbol** | **Entrez gene ID** | **Method** | **NGT**  **(N=7)** | **T2DM**  **(N=5)** | ***P*** |
| --- | --- | --- | --- | --- | --- |
| *STARD13* | 90627 | GCRMA | 7.92 ± 0.18 | 12.1 ± 0.82 | < 0.001 |
| *CPPED1* | 55313 | MAS5 | 6.25 ± 0.33 | 11.18 ± 1.14 | < 0.001 |
| *ABCC4* | 10257 | PLIER | 13.24 ± 2.12 | 30.84 ± 4.73 | 0.003 |
| *MOSC1* | 64757 | PLIER | 9.84 ± 0.9 | 17 ± 2.29 | 0.005 |
| *KCNIP4* | 80333 | PLIER | 14.01 ± 1.95 | 23.93 ± 2.67 | 0.012 |
| *ATP9B* | 374868 | MAS5 | 9.42 ± 1.05 | 18.11 ± 4.07 | 0.013 |
| *SNX29* | 92017 | MAS5 | 6.96 ± 0.71 | 4.58 ± 0.48 | 0.013 |
| *CDH23* | 64072 | PLIER | 8.59 ± 0.29 | 11.24 ± 1.05 | 0.017 |
| *CRBN* | 51185 | RMA | 98.58 ± 8.33 | 132.2 ± 6.36 | 0.020 |
| *POLR2C* | 5432 | RMA | 213.48 ± 7.69 | 179.84 ± 9.17 | 0.020 |
| *CLMN* | 79789 | RMA | 114.14 ± 5.69 | 143.84 ± 9.35 | 0.020 |
| *CP110* | 9738 | PLIER | 30.18 ± 3.56 | 45.11 ± 2.05 | 0.027 |
| *NPAS3* | 64067 | RMA | 15.74 ± 0.38 | 17.81 ± 0.8 | 0.027 |
| *C11orf71* | 54494 | MAS5 | 35.57 ± 3.87 | 24.55 ± 1.62 | 0.028 |
| *CA8* | 767 | MAS5 | 29.93 ± 4.77 | 14.66 ± 3.6 | 0.028 |
| *SYN2* | 6854 | MAS5 | 12.8 ± 2.31 | 22.35 ± 3.44 | 0.029 |
| *RBM7* | 10179 | MAS5 | 108.93 ± 12.99 | 58.33 ± 13.45 | 0.029 |
| *ESRRG* | 2104 | PLIER | 25.71 ± 2.15 | 37.81 ± 6.1 | 0.034 |
| *C20orf94* | 128710 | RMA | 15.09 ± 0.57 | 19.08 ± 1.83 | 0.036 |
| *FAM5C* | 339479 | MAS5 | 1.31 ± 0.09 | 2.14 ± 0.53 | 0.037 |
| *SCN1A* | 6323 | RMA | 7.34 ± 0.08 | 7.87 ± 0.26 | 0.043 |
| *LMO7* | 4008 | RMA | 15.46 ± 0.29 | 18.61 ± 1.63 | 0.043 |
| *ZNF282* | 8427 | GCRMA | 16.8 ± 1.3 | 13.52 ± 0.76 | 0.045 |
| *ST3GAL1* | 6482 | GCRMA | 35.2 ± 4.3 | 58.81 ± 13.92 | 0.046 |
| *CAMKK2* | 10645 | PLIER | 22.55 ± 4.18 | 35.82 ± 2.22 | 0.049 |
| *ARHGAP24* | 83478 | RMA | 16.41 ± 0.55 | 19.42 ± 1.47 | 0.049 |

1. **Healthy vs. Diabetic rat pancreas**

| **Gene Symbol** | **Entrez gene ID** | **Method** | **Healthy**  **(N=3)** | **Diabetic**  **(N=3)** | ***P*** |
| --- | --- | --- | --- | --- | --- |
| *Cdh23* | 114102 | PLIER | 43.33 ± 0.95 | 17.58 ± 1.1 | < 0.001 |
| *Tgfb3* | 25717 | PLIER | 162.3 ± 2.75 | 125.37 ± 2.42 | 0.001 |
| *Pqlc1* | 361352 | RMA | 268.68 ± 4.33 | 217.22 ± 3.25 | 0.001 |
| *Npffr1* | 64107 | PLIER | 137.43 ± 1.7 | 175.96 ± 4.54 | 0.001 |
| *Plekha1* | 361659 | MAS5 | 26.75 ± 3.19 | 84.23 ± 1.54 | 0.001 |
| *Znf282* | 297065 | PLIER | 60.32 ± 4.74 | 102.92 ± 1.04 | 0.002 |
| *Adnp2* | 307236 | PLIER | 9.61 ± 0.78 | 18.91 ± 0.76 | 0.002 |
| *Socs6* | 307200 | MAS5 | 127.83 ± 4.56 | 168.06 ± 3.08 | 0.002 |
| *Adarb2* | 117088 | MAS5 | 43.3 ± 4.62 | 88.62 ± 4.87 | 0.003 |
| *Nmu* | 63887 | MAS5 | 138.31 ± 9.47 | 222.45 ± 6.69 | 0.003 |
| *Gphn* | 64845 | MAS5 | 24.53 ± 0.58 | 41.26 ± 3.56 | 0.004 |
| *Nrxn1* | 60391 | RMA | 57.46 ± 0.95 | 49.59 ± 0.91 | 0.004 |
| *Tspan8* | 171048 | MAS5 | 49.86 ± 4.02 | 18.82 ± 2.69 | 0.004 |
| *Grm8* | 60590 | PLIER | 130.43 ± 7.15 | 91.99 ± 1.14 | 0.004 |
| *Klhl21* | 313743 | RMA | 106.71 ± 1.4 | 115.65 ± 0.56 | 0.005 |
| *Slc2a1* | 24778 | PLIER | 208.11 ± 10.48 | 134.07 ± 8.2 | 0.006 |
| *Smarcad1* | 312398 | RMA | 41.64 ± 1.24 | 51.88 ± 1.4 | 0.006 |
| *Kcnn2* | 54262 | MAS5 | 3.72 ± 0.2 | 11.36 ± 2.11 | 0.006 |
| *Gcom1* | 363091 | PLIER | 20.64 ± 3.78 | 64.73 ± 6.79 | 0.007 |
| *Map3k11* | 309168 | RMA | 259.79 ± 29.26 | 458.15 ± 6.91 | 0.007 |
| *Ptgds2* | 58962 | PLIER | 96.59 ± 8.87 | 59 ± 1.34 | 0.007 |
| *Tbc1d23* | 304019 | PLIER | 29.33 ± 0.94 | 37.53 ± 1.4 | 0.007 |
| *Kcng2* | 307234 | RMA | 123.77 ± 5.09 | 90.13 ± 4.4 | 0.007 |
| *Syn2* | 29179 | PLIER | 38.76 ± 0.95 | 25 ± 2.15 | 0.008 |
| *Crk* | 54245 | RMA | 44.31 ± 1.61 | 37.09 ± 0.28 | 0.008 |
| *Cast* | 25403 | RMA | 48.71 ± 1.23 | 41.02 ± 0.99 | 0.008 |
| *Ddrgk1* | 296162 | RMA | 848.52 ± 43.72 | 666.41 ± 2.78 | 0.009 |
| *Snf8* | 287645 | PLIER | 256.89 ± 16.09 | 384.14 ± 23.66 | 0.009 |
| *Mpv17l* | 360463 | RMA | 148.29 ± 5.29 | 124.49 ± 1.54 | 0.010 |
| *Tmem131* | 316335 | RMA | 176.67 ± 5.98 | 128.94 ± 7.64 | 0.010 |
| *Cadps* | 26989 | RMA | 78.17 ± 1.21 | 46.65 ± 5.34 | 0.011 |
| *LOC690745* | 690745 | PLIER | 142.57 ± 8.33 | 99.09 ± 5.82 | 0.011 |
| *Kcnj6* | 25743 | PLIER | 58.87 ± 4.28 | 87.04 ± 4.14 | 0.012 |
| *Mcm3* | 316273 | RMA | 142.44 ± 6.46 | 112.84 ± 3.69 | 0.014 |
| *Svop* | 171442 | PLIER | 70.82 ± 1.37 | 50.28 ± 4.06 | 0.015 |
| *Tbc1d1* | 360937 | MAS5 | 104.36 ± 6.31 | 69.64 ± 5.69 | 0.015 |
| *Magi2* | 113970 | PLIER | 158.2 ± 12.84 | 224.91 ± 4.93 | 0.016 |
| *LOC679869* | 679869 | PLIER | 55.33 ± 5.18 | 80.67 ± 1.42 | 0.016 |
| *Lmo7* | 361084 | MAS5 | 125.09 ± 18.31 | 50.11 ± 9.45 | 0.016 |
| *Polr2c* | 361365 | RMA | 207.14 ± 4.9 | 187.24 ± 1.89 | 0.017 |
| *Lrfn2* | 316205 | PLIER | 23.33 ± 4.29 | 49.06 ± 1.35 | 0.017 |
| *Cpped1* | 302890 | PLIER | 122.82 ± 1.83 | 158.06 ± 9.64 | 0.017 |
| *Col4a1* | 290905 | RMA | 208.74 ± 8.79 | 174.49 ± 3.02 | 0.017 |
| *LOC493574* | 493574 | RMA | 21.03 ± 0.45 | 19 ± 0.29 | 0.018 |
| *Kalrn* | 84009 | PLIER | 172.38 ± 6.47 | 216.42 ± 10.17 | 0.019 |
| *Gabra5* | 29707 | PLIER | 35.33 ± 1.47 | 51.11 ± 4.84 | 0.021 |
| *Sc4mol* | 140910 | RMA | 175.29 ± 8.03 | 220.7 ± 9.52 | 0.021 |
| *Dtd1* | 362227 | PLIER | 261.83 ± 6.42 | 297.93 ± 7.78 | 0.022 |
| *Gnaq* | 81666 | RMA | 49.93 ± 1.92 | 62.05 ± 3.02 | 0.024 |
| *Foxp1* | 297480 | RMA | 36.45 ± 1.1 | 43.5 ± 1.77 | 0.025 |
| *Grid2* | 79220 | PLIER | 9.35 ± 1.47 | 4.63 ± 0.63 | 0.027 |
| *Notch2* | 29492 | PLIER | 101.81 ± 0.46 | 110.92 ± 2.8 | 0.028 |
| *RGD1310423* | 361112 | RMA | 105.3 ± 5.48 | 87.33 ± 1.74 | 0.029 |
| *Sart3* | 304582 | RMA | 107.22 ± 5.34 | 130.59 ± 4.3 | 0.029 |
| *Erap1* | 80897 | MAS5 | 60.83 ± 2.94 | 40.3 ± 4.9 | 0.031 |
| *Kif2a* | 84391 | MAS5 | 44.1 ± 3.19 | 62.91 ± 4.93 | 0.031 |
| *Inpp5d* | 54259 | RMA | 251.4 ± 13.82 | 206.76 ± 5.09 | 0.031 |
| *Slc6a3* | 24898 | RMA | 224.77 ± 4.18 | 206.9 ± 4.05 | 0.037 |
| *Arid3b* | 367092 | RMA | 217.97 ± 7.05 | 248.4 ± 7.33 | 0.040 |
| *Msra* | 29447 | RMA | 87.34 ± 3.63 | 54.5 ± 8.95 | 0.040 |
| *Pcsk5* | 116548 | RMA | 56.18 ± 1.27 | 48.28 ± 2.31 | 0.043 |
| *Kcns3* | 83588 | MAS5 | 35.92 ± 4.15 | 25.37 ± 1.26 | 0.044 |
| *Ngdn* | 305887 | PLIER | 200.38 ± 18.93 | 146.8 ± 8.99 | 0.048 |
| *Rad51ap1* | 689055 | RMA | 38.48 ± 0.78 | 34.6 ± 1.13 | 0.049 |

1. **NGT vs. T2DM human skeletal muscle**

| **Gene Symbol** | **Entrez gene ID** | **Method** | **NGT**  **(N=17)** | **T2DM**  **(N=18)** | ***P*** |
| --- | --- | --- | --- | --- | --- |
| *TNFSF10* | 8743 | PLIER | 56.6 ± 2.96 | 45.97 ± 2.28 | 0.005 |
| *CRK* | 1398 | MAS5 | 45.67 ± 2.52 | 35.75 ± 2.42 | 0.011 |
| *PPIH* | 10465 | RMA | 46.57 ± 2.21 | 38.82 ± 2.02 | 0.011 |
| *SC4MOL* | 6307 | RMA | 16.82 ± 0.6 | 15.11 ± 0.37 | 0.017 |
| *DIMT1L* | 27292 | PLIER | 64.53 ± 4.98 | 51.05 ± 3.68 | 0.018 |
| *RAMP1* | 10267 | GCRMA | 83.33 ± 9.28 | 57.8 ± 5.71 | 0.022 |
| *SAMD4A* | 23034 | PLIER | 109.45 ± 14.79 | 64.17 ± 14.53 | 0.023 |
| *C11orf71* | 54494 | PLIER | 45.93 ± 2.17 | 38.44 ± 2.31 | 0.024 |
| *CTNNA2* | 1496 | MAS5 | 5.8 ± 2.89 | 11.44 ± 3.02 | 0.025 |
| *CRBN* | 51185 | GCRMA | 122.49 ± 12.52 | 91.95 ± 7.13 | 0.031 |
| *SNF8* | 11267 | MAS5 | 56.63 ± 4.52 | 39.65 ± 5.12 | 0.031 |
| *SPAM1* | 6677 | GCRMA | 6.7 ± 0.62 | 5.7 ± 0.12 | 0.033 |
| *SLC20A2* | 6575 | MAS5 | 47.07 ± 7.24 | 74.16 ± 9.49 | 0.039 |
| *SOCS6* | 9306 | MAS5 | 8.19 ± 1.43 | 13.26 ± 1.6 | 0.041 |
| *ELF1* | 1997 | PLIER | 26.95 ± 1.47 | 22.67 ± 1.34 | 0.046 |
| *PHACTR1* | 221692 | RMA | 27.88 ± 0.57 | 30.93 ± 1.69 | 0.046 |
| *RORA* | 6095 | PLIER | 45.62 ± 3.77 | 34.86 ± 2.67 | 0.048 |
| *KIAA0494* | 9813 | GCRMA | 20.98 ± 0.05 | 21.28 ± 0.14 | 0.048 |

1. **Healthy vs. Diabetic mice skeletal muscle**

| **Gene Symbol** | **Entrez gene ID** | **Method** | **Healthy**  **(N=8)** | **Diabetic**  **(N=7)** | ***P*** |
| --- | --- | --- | --- | --- | --- |
| *Ramp1* | 51801 | MAS5 | 111.8 ± 15.29 | 62.83 ± 5.97 | 0.005 |
| *Arid3b* | 56380 | MAS5 | 10.45 ± 1.19 | 17.33 ± 1.61 | 0.006 |
| *Gnaq* | 14682 | PLIER | 18.02 ± 0.6 | 15.16 ± 0.71 | 0.007 |
| *Notch2* | 18129 | RMA | 39.87 ± 0.81 | 43.13 ± 0.75 | 0.011 |
| *Kcnj11* | 16514 | PLIER | 64.98 ± 3.7 | 45.02 ± 6.99 | 0.021 |
| *Kif11* | 16551 | RMA | 9.2 ± 0.18 | 8.49 ± 0.22 | 0.024 |
| *Adcy2* | 210044 | MAS5 | 43.09 ± 6.67 | 62.96 ± 4.29 | 0.027 |
| *Pdia3* | 14827 | MAS5 | 18.86 ± 1.43 | 13.52 ± 1.88 | 0.031 |
| *Grm8* | 14823 | PLIER | 25.62 ± 2.41 | 14.94 ± 3.6 | 0.031 |
| *Sc4mol* | 66234 | MAS5 | 2.75 ± 0.72 | 4.88 ± 0.43 | 0.036 |
| *Nmu* | 56183 | PLIER | 23.16 ± 2 | 16.63 ± 2.01 | 0.044 |
| *Wsb2* | 59043 | GCRMA | 70.69 ± 7.36 | 51.01 ± 5.32 | 0.044 |
| *Prpf31* | 68988 | MAS5 | 31.66 ± 3.67 | 20.95 ± 2.8 | 0.044 |
| *Stac* | 20840 | MAS5 | 5.82 ± 1.34 | 10.25 ± 1.16 | 0.045 |
| *Tgfbr3* | 21814 | PLIER | 42.51 ± 1.63 | 48.17 ± 2.04 | 0.045 |
| *Eya4* | 14051 | PLIER | 0.49 ± 0.7 | 0.02 ± 0.49 | 0.046 |
| *Cadps* | 27062 | MAS5 | 4.46 ± 0.61 | 3.22 ± 0.19 | 0.048 |
| *Gcom1* | 102371 | PLIER | 0.13 ± 0.51 | 2.28 ± 0.94 | 0.048 |
| *Galnt10* | 171212 | GCRMA | 5.28 ± 0.01 | 5.36 ± 0.04 | 0.049 |

1. **Zucker diabetic fatty rat (ZDF) vs. Zucker lean control (ZLC)**

| **Gene Symbol** | **Entrez GeneID** | **Tissue** | **Age** | **Expression ratios (ZDF/ZLC)**  **(N=4)^§^** |
| --- | --- | --- | --- | --- |
| *Col4a1* | 290905 | Adipose | 6 weeks | 1.58 |
| *Slc2a1* | 24778 | Adipose | 12 weeks | 0.46 |
| *Pdia3* | 29468 | Adipose | 12 weeks | 0.46 |
| *Foxp1* | 297480 | Adipose | 12 weeks | 0.50 |
| *Igf2bp2* | 303824 | Adipose | 12 weeks | 0.65 |
| *Lmo7* | 361084 | Adipose | 12 weeks | 1.55 |
| *Stard13* | 498130 | Adipose | 12 weeks | 1.73 |
| *Chd2* | 308738 | Adipose | 12 weeks | 1.99 |
| *LOC690745* | 690745 | Adipose | 12 weeks | 2.24 |
| *Col4a1* | 290905 | Adipose | 12 weeks | 2.32 |
| *Slc25a26* | 362403 | Liver | 6 weeks | 1.67 |
| *Slc2a1* | 24778 | Liver | 6 weeks | 1.78 |
| *Pdia3* | 29468 | Liver | 12 weeks | 0.55 |
| *Sc4mol* | 140910 | Liver | 12 weeks | 0.60 |
| *Cast* | 25403 | Liver | 12 weeks | 0.62 |
| *Il6ra* | 24499 | Liver | 12 weeks | 0.64 |
| *Stard13* | 498130 | Liver | 12 weeks | 1.53 |
| *Erlin1* | 293939 | Liver | 12 weeks | 1.65 |
| *Ankrd15* | 309429 | Muscle | 6 weeks | 0.58 |
| *Sc4mol* | 140910 | Muscle | 6 weeks | 0.62 |
| *Foxp1* | 297480 | Muscle | 6 weeks | 0.62 |
| *Gap43* | 29423 | Muscle | 12 weeks | 0.22 |
| *Tmem131* | 316335 | Muscle | 12 weeks | 0.57 |
| *LOC690745* | 690745 | Muscle | 12 weeks | 0.63 |
| *Fam45a* | 308009 | Muscle | 12 weeks | 0.65 |
| *Actn1* | 81634 | Muscle | 12 weeks | 1.53 |
| *Chd2* | 308738 | Muscle | 12 weeks | 1.81 |
| *Brunol4* | 307540 | Muscle | 12 weeks | 1.86 |
| *Erlin1* | 293939 | Muscle | 12 weeks | 2.05 |

Values are mean ± SEM, *P*-values refer to a two-tailed Student's *t*-test with equal variance. ^§^Expression ratios are shown as normalized mean signal intensity of a gene of ZDF to ZLC.

**Abbreviations**: NGT, normal glucose tolerance; T2DM, type 2 diabetes mellitus; GC-RMA, GC-content robust multi-array average; PLIER, probe logarithmic intensity error; RMA, robust multi-array average.

**Supplemental Table S2**: T2DM and related traits associated genes for which expression levels in pancreas, skeletal muscle, adipose tissue or liver were altered in diabetes as compared to controls

1. **NGT vs. T2DM human pancreatic islets**

| **Gene Symbol** | **Entrez gene ID** | **Method** | **NGT**  **(N=7)** | **T2DM**  **(N=5)** | ***P*** |
| --- | --- | --- | --- | --- | --- |
| *HNF1B* | 6928 | MAS5 | 142.36 ± 18.49 | 293.57 ± 57.02 | 0.009 |
| *THADA* | 63892 | MAS5 | 29.48 ± 2.36 | 40.54 ± 4.31 | 0.039 |

1. **Healthy vs. Diabetic rat pancreas**

| **Gene Symbol** | **Entrez gene ID** | **Method** | **Healthy**  **(N=3)** | **Diabetic**  **(N=3)** | ***P*** |
| --- | --- | --- | --- | --- | --- |
| *Hnf1b* | 25640 | PLIER | 91.71 ± 2.19 | 63.92 ± 0.59 | < 0.001 |
| *Wfs1* | 83725 | PLIER | 84.72 ± 5.8 | 51.21 ± 2.09 | 0.003 |
| *Tspan8* | 171048 | MAS5 | 49.86 ± 4.02 | 18.82 ± 2.69 | 0.004 |
| *Kcnq1* | 84020 | MAS5 | 181.99 ± 18.6 | 341.42 ± 19.42 | 0.005 |
| *Hhex* | 79237 | PLIER | 470.42 ± 12.65 | 552.59 ± 10.99 | 0.008 |
| *LOC679869* | 679869 | PLIER | 55.33 ± 5.18 | 80.67 ± 1.42 | 0.016 |
| *Notch2* | 29492 | PLIER | 101.81 ± 0.46 | 110.92 ± 2.8 | 0.028 |

1. **NGT vs. T2DM human skeletal muscle**

| **Gene Symbol** | **Entrez gene ID** | **Method** | **NGT**  **(N=17)** | **T2DM**  **(N=18)** | ***P*** |
| --- | --- | --- | --- | --- | --- |
| *HNF1B* | 6928 | PLIER | 57.08 ± 3.2 | 48.65 ± 2.61 | 0.045 |

1. **Healthy vs. Diabetic mice skeletal muscle**

| **Gene Symbol** | **Entrez gene ID** | **Method** | **Healthy**  **(N=8)** | **Diabetic**  **(N=7)** | ***P*** |
| --- | --- | --- | --- | --- | --- |
| *Notch2* | 18129 | RMA | 39.87 ± 0.81 | 43.13 ± 0.75 | 0.011 |
| *Kcnj11* | 16514 | PLIER | 64.98 ± 3.7 | 45.02 ± 6.99 | 0.021 |
| *Wfs1* | 22393 | RMA | 135.63 ± 5.45 | 117.38 ± 5.79 | 0.034 |
| *Irs1* | 16367 | MAS5 | 5.85 ± 0.7 | 3.33 ± 0.61 | 0.036 |

1. **Zucker diabetic fatty rat (ZDF) vs. Zucker lean control (ZLC)**

| **Gene Symbol** | **Entrez GeneID** | **Tissue** | **Age** | **Expression ratios (ZDF/ZLC)**  **(N=4)^§^** |
| --- | --- | --- | --- | --- |
| *Igf2bp2* | 303824 | Adipose | 12 weeks | 0.65 |
| *Pparg* | 25664 | Adipose | 12 weeks | 2.91 |
| *Pparg* | 25664 | Liver | 6 weeks | 1.84 |

Values are mean ± SEM, *P*-values refer to a two-tailed Student's *t*-test with equal variance. ^§^Expression ratios are shown as normalized mean signal intensity of a gene of ZDF to ZLC.

**Abbreviations**: NGT, normal glucose tolerance; T2DM, type 2 diabetes mellitus; GC-RMA, GC-content robust multi-array average; PLIER, probe logarithmic intensity error; RMA, robust multi-array average.
